# CoNutriNet: a dual-branch architecture with DenseNet and graph-enhanced attention network for coffee nutrient deficiency classification

**DOI:** 10.3389/fpls.2026.1827047

**Published:** 2026-06-12

**Authors:** Dhivyaa P. V., Karthik R., Vishnu Dhananjai S., Dhanvasanth R., Alden Jenish S., Suganthi K.

**Affiliations:** 1 School of Electronics Engineering, Vellore Institute of Technology, Chennai, India; 2 Centre for Cyber-Physical Systems (CCPS), Vellore Institute of Technology, Chennai, India

**Keywords:** attention mechanism, coffee leaves, convolutional neural networks, DenseNet, graph convolution, nutrient deficiency

## Abstract

**Introduction:**

Nutrient deficiencies in coffee plants significantly impact bean quality and yield, making timely detection crucial for successful cultivation. Current assessment methods rely on manual inspection, which is labor-intensive and time-consuming, posing challenges for large-scale field management. This approach often results in inconsistent evaluations and delayed interventions.

**Methods:**

This study presents CoNutriNet, an automated deep learning architecture that integrates DenseNet121 with a novel Graph-Enhanced Attention Feature Network (GEAFNet) for classifying nutrient deficiencies in coffee leaves. DenseNet121 provides deep hierarchical and regional feature representation, while GEAFNet captures local, fine-grained spatial features through Inception, Ghost, and Efficient Channel Attention (ECA) modules. Furthermore, a Graph Convolutional Network (GCN) is included to model spatial dependencies and structural variations between leaf regions. Feature representations from both pathways are concatenated and refined using a Coordinate Attention (CA) module to enhance discriminative capability.

**Results:**

Evaluation on the CoLeaf dataset demonstrates that CoNutriNet achieves an accuracy of 94.5%. The integration of lightweight attention mechanisms, dense connectivity, and graph-based modeling improves both performance and computational efficiency.

**Conclusion:**

These results indicate that CoNutriNet achieves and efficient performance in nutrient deficiency detection in coffee crops, highlighting its potential for deployment in agricultural environments to support precision farming and optimize yield.

## 1 Introduction

Coffee is extensively derived from the Coffea arabica and Coffea robusta species. It is one of the most consumed beverages in the world and is an essential commodity to millions of farmers. The high-quantity production of these coffee plants requires them to maintain optimal health conditions and sustainable environments. The importance of nutrients that are fundamental to the health of the coffee plants has been emphasized in recent studies. These nutrients are necessary in the main sites in the plants for photosynthesis and energy production required for coffee bean development. Therefore, understanding their role has become a vital criterion in evaluating the overall plant’s growth, health, and productivity.

Nutrients are important substances that plants absorb to support their growth, development, and reproduction. In plant leaves, these nutrients are classified into macronutrients and micronutrients. They also play an important role in enzyme function, photosynthesis, and maintaining an intact cell structure. An adequate supply of these nutrients supports strong plant growth and aids plants in coping with environmental stress and producing high yields. The absence of these nutrients can be identified through noticeable signs such as stunted growth, yellowing of leaves, and reduced productivity. They also reduce the quality of the coffee beans produced, significantly affecting the harvests (Diriba, 2022). Hence, early and accurate detection of these deficiencies is vital for improving crop health.

Conventionally, the methods of identification rely on manual visual observation or chemical testing for diagnosing the deficiencies in coffee leaves. However, they are slow, labor-intensive, require expert knowledge, and are susceptible to subjectivity. These factors lead to inconsistent results and increase the error rates in detection. Additionally, they also render these manual methods impractical and difficult to practice on modern large-scale coffee plantations (Patel et al., 2025). The increasing importance of precision agriculture has made the need for automation in detecting nutrient deficiency. This has paved the way for the development of intelligent automated systems that integrate Computer Vision (CV) and Artificial Intelligence (AI) that help provide consistent and scalable solutions. Studies utilize Machine Learning (ML) models to perform image processing and help in monitoring plant health and disease classification. Recent advances in DL systems have changed how experts monitor plant health. It provides efficient automated systems that allow quick and accurate detection of nutrient deficiencies from leaf images (Chen et al., 2020). Studies utilize Convolutional Neural Networks (CNNs) and transformer-based architectures, which are effective in recognizing subtle visual features and don’t require manual feature engineering (Vassallo-Barco et al., 2017; Bera et al., 2024). It is also important to ensure that the model achieves efficient performance under different leaf shapes, orientations, and overlapping nutrient symptoms (Tuesta-Monteza et al., 2023; Ramirez-Builes et al., 2024). Additionally, there exists a necessity to develop lightweight and computationally efficient models that can be employed in resource-constrained locations. This study introduces a lightweight attention-based DL model focused on improving the classification of nutrient deficiencies in coffee leaves. The key contributions made in the study are the following:

- A dual-track architecture that combines DenseNet-121 to capture global leaf features with a lightweight GEAFNet branch that concentrates on local fine-grained details.
- The GEAFNet utilizes Inception and Ghost modules along with graph-based learning and attention mechanisms. This design helps the model focus on important areas, such as tips, margins, and veins, where nutrient symptoms usually appear.
- Integration of the CA mechanism, which combines global and local attention features and achieves efficient performance while maintaining computational efficiency.

## 2 Related works

The automated detection of nutrient deficiencies in coffee plants has evolved significantly through classical ML and DL systems. This section presents the existing systems and approaches for nutrient deficiency diagnosis.

### 2.1 Machine learning approaches

ML models identify nutrient deficiencies in coffee leaves by analyzing images to extract features such as color, texture, and shape. The extracted features are processed to classify the nutritional condition and detect any signs of imbalance in the leaves. These models find patterns in the surface texture and discoloration to provide accurate diagnoses and support early detection of nutrient-related issues.

Balamurugan et al. induced nutrient deficiencies such as nitrogen, phosphorus, and potassium in the plants and measured leaf reflectance using a spectroradiometer at specific growth stages (Balamurugan et al., 2024). The study identified nutrient-sensitive wavelengths and utilized ML and spectral analysis for improved detection. Sabzi et al. utilized Artificial Neural Networks (ANNs) and partial least squares regression for nutrient detection. The model was enhanced by particle swarm optimization for nitrogen estimation with hyperspectral imaging (Sabzi et al., 2021). Barman et al. acquired images of different leaf types as tender, immature, and mature using a smartphone (Barman and Choudhury, 2022). The study extracted color features from the images and quantified the actual chlorophyll content using spectrophotometer instrumentation as ground truth. The study also implemented a linear regression model and an ANN feed-forward network to assess chlorophyll content. The models were trained using the Levenberg-Marquardt and scaled conjugate gradient backpropagation algorithms.

Sekerli et al. measured the nutrient contents in plant leaves by utilizing a regression model combined with Fourier transform near-infrared spectroscopy (Sekerli et al., 2024). Chwastyk et al. performed fieldwork on the samples where the study obtained hyperspectral data using drones, ground-based FieldSpec sensors (Panek-Chwastyk et al., 2025). The study standardized and pre-processed the data to train a feedforward neural network. The study also presented the importance of spectral features that were determined using gradient-based analyses to assess individual features in the spectral bands used for predicting phosphorus.

Motta et al. investigated ML algorithms integrated with feature selection mechanisms for nutrient diagnosis in plants (Motta et al., 2024). Mahendran et al. incorporated Internet of Things (IoT) devices and ML algorithms to identify nutrient deficiencies in leaves, which advanced remote sensing to be real-time (Mahendran et al., 2022). Rizal et al. applied CNN as feature extractors in the workflow of ML to classify nutrient deficiencies in plant leaves (Rizal et al., 2022). Sharma et al. used vision transformers and ML algorithms to detect macro- and micronutrient deficiencies in the leaves. The study leveraged the benefits of both traditional and newer methods (Sharma and Vishwakarma, 2024). Shadrach et al. implemented a transfer learning-based hybrid ML-DL solution for improved performance of the nutrient deficiencies classification (Shadrach et al., 2023).

Despite promising results and improvements in predictability, support systems for plant nutrient deficiency detection still require a significant dependence on manual feature engineering and contend with the challenge of image variability. The DL models have gained traction as they can automatically detect the hierarchical levels of features in an image and generalize the results across plant species and imaging conditions. Since DL models can automatically extract hierarchical features and generalize across a variety of plant species and imaging conditions, recent studies have increasingly adopted them.

### 2.2 Deep learning approaches

DL has demonstrated effectiveness over traditional ML approaches in identifying and classifying nutrient deficiencies in plant leaves. Unlike ML models, which require manual feature engineering, DL models can extract patterns and intricate features directly from raw data. This enables DL models to efficiently process large and complex datasets, learning subtle indicators of nutrient deficiencies that traditional methods may overlook. Sunitha et al. introduced a skip-connected CNN model to identify boron and iron deficiency in the leaves (Sunitha et al., 2024). Ali et al. employed a CNN with four convolutional layers to classify potassium, magnesium, nitrogen, and phosphorus deficiencies on a custom dataset (Ali et al., 2022). Gallegos et al. utilized different ML algorithms along with CNNs for nitrogen estimation (Gallegos et al., 2025). Sona et al. employed a DL-based approach for detecting nutrient deficiencies in plant leaves using images captured by mobile devices (Haris et al., 2023). A pre-trained lightweight deep CNN was utilized to extract features from the images and identify regions affected by nutrient deficiencies, classifying leaves as either nutrient-deficient or healthy.

Han et al. implemented the combination of old and young leaf samples to classify nutrient deficiencies (Myo Han et al., 2020). The study extracted features from these leaves using a pre-trained ResNet50 CNN and then employed classification using logistic regression, SVM, and Multilayer Perceptron (MLP) models. Nirasha et al. annotated the leaf images and trained two models: Mask Region-based CNN (Mask R-CNN) for classification and segmentation and You Only Look Once (YOLO) for rapid deficiency classification (Jayasiri et al., 2023).

Buhawe et al. implemented an Xception-based CNN for maturity and nutrient grading in plants grown in hydroponics (Buhawe et al., 2022). Wang et al. utilized hyperspectral imaging along with CNNs for NPK stress classification, showing resilience in field environments (Wang et al., 2024). Liu et al. employed a multi-scale feature fusion network for effective handling of leaf-scale variations in leaf nutrient diagnosis (Liu et al., 2024). Anita et al. introduced a deep neural network incorporated with feature selection and optimization strategies to improve the classification performance compared to traditional methods (Anitha et al., 2022). Laurence et al. evaluated ResNet-50, VGG-16, and InceptionV3 CNN models for identifying nitrogen, potassium, and phosphorus deficiencies using image analysis (Navarro et al., 2023). Xu et al. conducted a study to diagnose nutrient deficiencies using image-based DL methods (Xu et al., 2020). The sample images were collected through hydroponic experiments, covering full nutrition and ten different nutrient deficiencies. The study fine-tuned four state-of-the-art deep CNNs, Inception-v3, ResNet50, NasNet-Large, and DenseNet121, using transfer learning and compared this study’s performance to traditional ML methods. Kavitha et al. implemented a customized CNN with transfer learning for multi-nutrient deficiency detection (Kavitha and Chinnaiah, 2025). The methodology involves image preprocessing using improved Wiener filtering, segmentation with a modified U-Net model, and final identification using the proposed system.

Talukder et al. utilized five pre-trained CNN models: InceptionV3, InceptionResNetV2, DenseNet121, DenseNet169, and DenseNet201 (Talukder and Sarkar, 2023). The models with best performance, DenseNet169, DenseNet201, and InceptionV3, were combined in a weighted ensemble for nutrient deficiency classification. Sudhakar et al. also employed an ensemble DL approach for detecting micronutrient deficiencies (Muthusamy and Ramu, 2024). The study trained and evaluated six modified transfer learning models and created ensemble classifiers by averaging the predictions of top-performing models.

Charan et al. implemented a dual-track DL framework for classifying nutrient deficiencies in coffee leaves (Sai Charan et al., 2025). This study integrated DenseNet to extract global, hierarchical features with a custom CNN for local feature extraction. The model incorporates Inception modules, ECA, Ghost modules, and channel shuffling to enhance feature representation and computational efficiency. Venkatesh et al. applied ensemble transfer learning to detect nutrient deficiencies and yield losses in crops (Venkatesh and Naik, 2024). Liao et al. designed a hybrid CNN-LSTM model that integrated spatial and temporal patterns in plant images, achieving accurate detection of nitrogen, phosphorous, and potassium deficiencies (Liao et al., 2024). Bera et al. created a custom CNN integrated with graph convolutional networks for capturing spatial relationships in nutrient-deficient plant leaves (Bera et al., 2024). Yogabalajee et al. evaluated different DL models, including ResNet50, Vision Transformers, ConvNeXt, and ConvNeXtV2, for detecting and classifying leaf diseases from images (Yogabalajee and Kaliappan, 2023). The study introduced a modified ConvNeXtV2 model, optimized with a Swin optimizer for an accurate and automated disease detection system. Amudha et al. employed a capsule network with contextual attention routing to classify deficiencies such as nitrogen, phosphorus, and potassium (Amudha and Brindha, 2024). The model uses convolutional layers, capsule layers, and an attention-based routing mechanism for efficient classification. In summary, these approaches demonstrate the efficiency of DL-based systems for nutrient deficiency classification in plant leaves. However, there still exist challenges, underscoring the need for further research to develop efficient model architectures.

### 2.3 Research gaps

This study aims to address limitations in the existing methods of leaf nutrient deficiency classification. The identified research gaps are:

- The majority of the currently available solutions overlook important regions of coffee leaves, which are important indicators of nutrient stress. These methods limit the model’s ability to learn both local and global features. Hence, models may not fully leverage the visual nutrient deficiency patterns in the coffee leaves.
- Most of the current studies often perform a feature extraction process using generic CNN architectures. This may hinder the model from capturing structural interrelationships from the coffee leaves that are helpful for improved performance.
- Incorporating complex mechanisms and large deep networks demands significant computational requirements and may limit the model’s applicability in resource-constrained fields. This renders the model ineffective and difficult to employ for monitoring large-scale coffee farms in real-time where low-cost and scalable solutions are required.

### 2.4 Research contributions

Unlike existing dual-branch or graph-based models, the proposed architecture introduces a structured integration of multi-scale patch-based graph modeling with lightweight attention mechanisms. The network is specifically tailored to capture spatially distributed nutrient deficiency patterns in coffee leaves. The contributions made in the study are:

- Multi-level feature fusion is achieved through the use of complementary cues at various scales. The proposed system leverages DenseNet to extract global features and GEAFNet to capture fine-grained feature representations and intermediate regional patterns. This design allows the model to learn both local detail and global structural patterns that contribute to the nutrient deficiency classification.
- Structural and spatial dependencies among the leaf regions are captured using the graph-based representation incorporated within the architecture. This enables the model to focus on the sensitive areas and important relations between leaf regions.
- The proposed architecture employs lightweight attention-based concatenation through ECA and CA mechanisms. This helps the model identify relevant spatial coordinates and responsive channels with low computational requirements.

## 3 Proposed network

**Figure 1:**
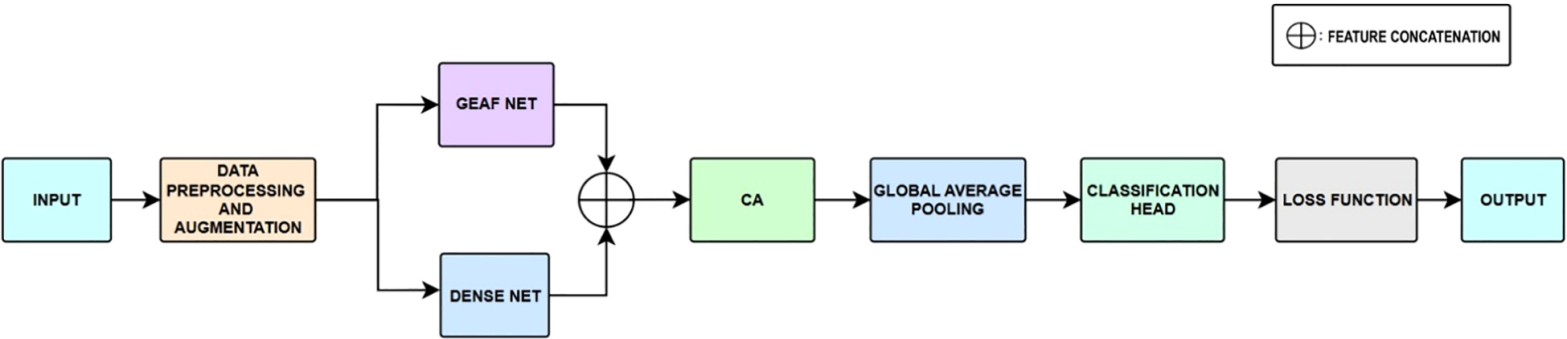
Structural design of the CoNutriNet.

The proposed CoNutriNet utilizes a dual-branch architecture for classifying nutrient deficiency in coffee leaves. DenseNet-121 is used to capture global hierarchical features in one branch, and a custom-designed network, GEAFNet, is used to capture local fine-grained features. DenseNet provides broader structural representations, while GEAFNet increases local fine-grained representation via Inception and Ghost modules and is further refined using ECA. In order to capture spatial dependencies among leaf subregions, GCN layers with attention are applied to multi-scale patch representations. The outputs from both branches are concatenated into a unified feature space and enhanced using the CA module. The fused features are passed to a global average pooling layer and then to a fully connected layer for classification. **Figure 1** illustrates the overview of the proposed system.

### 3.1 DenseNet-121

In the proposed model, DenseNet-121 is the global feature extraction track. DenseNet connects each layer to all the subsequent layers, allowing the visual patterns of veins and margins to propagate to higher layers to determine a higher-level semantic cue (Huang et al., 2017). For a dense block of L layers, the total number of direct connections is given by Equation 1.

(1) Connections=L(L+1)/2

The high degree of connectivity enables feature reuse and redundancy reduction so that it alleviates the vanishing gradient problem and improves convergence. Each layer of a dense block transforms the input/output by concatenating all feature maps from previous layers together to ensure that low-level and high-level information are maintained. For inputs, the output of the L^th^ layer is given, where it represents a composite operation of Batch Normalization (BN), ReLU activation, and convolution. This mechanism enables DenseNet-121 to combine structural vein-level details with overall shape-based information, which is crucial for identifying nutrient imbalances in coffee leaves. A key property of DenseNet is the growth rate k, which determines how many new feature maps each layer contributes. If the input to a dense block has F_0_​ feature maps, then after L layers, the number of output feature maps is given by Equation 2.

(2) $$F_{L}=F_{0}+k\cdot L$$

Transition layers are used to compress the feature maps for computational efficiency between blocks of dense feature maps. Each transition layer uses a 1×1 convolution to reduce the number of channels and a 2×2 average pooling to down-sample spatially. Furthermore, DenseNet employs bottleneck layers within each block to reduce parameters while maintaining representation power. Each bottleneck applies a 1×1 convolution followed by a 3×3 convolution. It improves memory efficiency and ensures that the extracted global features remain compact but highly informative. Finally, the global feature representation output from DenseNet-121 is denoted as per Equation 3.

(3) $$F_{global}=\phi_{\text{DenseNet}-121}\left(I\right)$$

where I is the input image of a coffee leaf and Φ is the complete DenseNet feature extraction algorithm. Within the proposed framework, these global features enhance the fine-grained local and regional features extracted using GEAFNet, leading to a detailed view of patterns involving the nutrient deficiencies.

### 3.2 Graph-enhanced efficient attention feature network

The objective of the GEAFNet is to enhance nutrient deficiency classification using efficient convolutional feature extraction in combination with a graph-based module. The architecture of the model is built to learn both local patterns and relational information from coffee leaf images to detect deficiencies.

Firstly, the Ghost module offers some reduction in computational requirements by producing a larger quantity of feature maps from a smaller quantity of intrinsic feature maps. This lightweight strategy allows for the early layers of the network to leverage the features with minor demand on computational resources. Subsequently, the inception block learns multi-scale information from parallel convolutions with diverse kernel sizes, allowing the model to encode fine-textural changes to leaves while simultaneously learning broader structures. To increase discriminative capacity, the ECA module provides an adaptive way to strengthen informative channels while still staying at the same dimensionality. As a result, the design allows the network to focus on important visual cues to nutrient deficiency, such as vein clarity, mottling, or discoloration.

**Figure 2:**
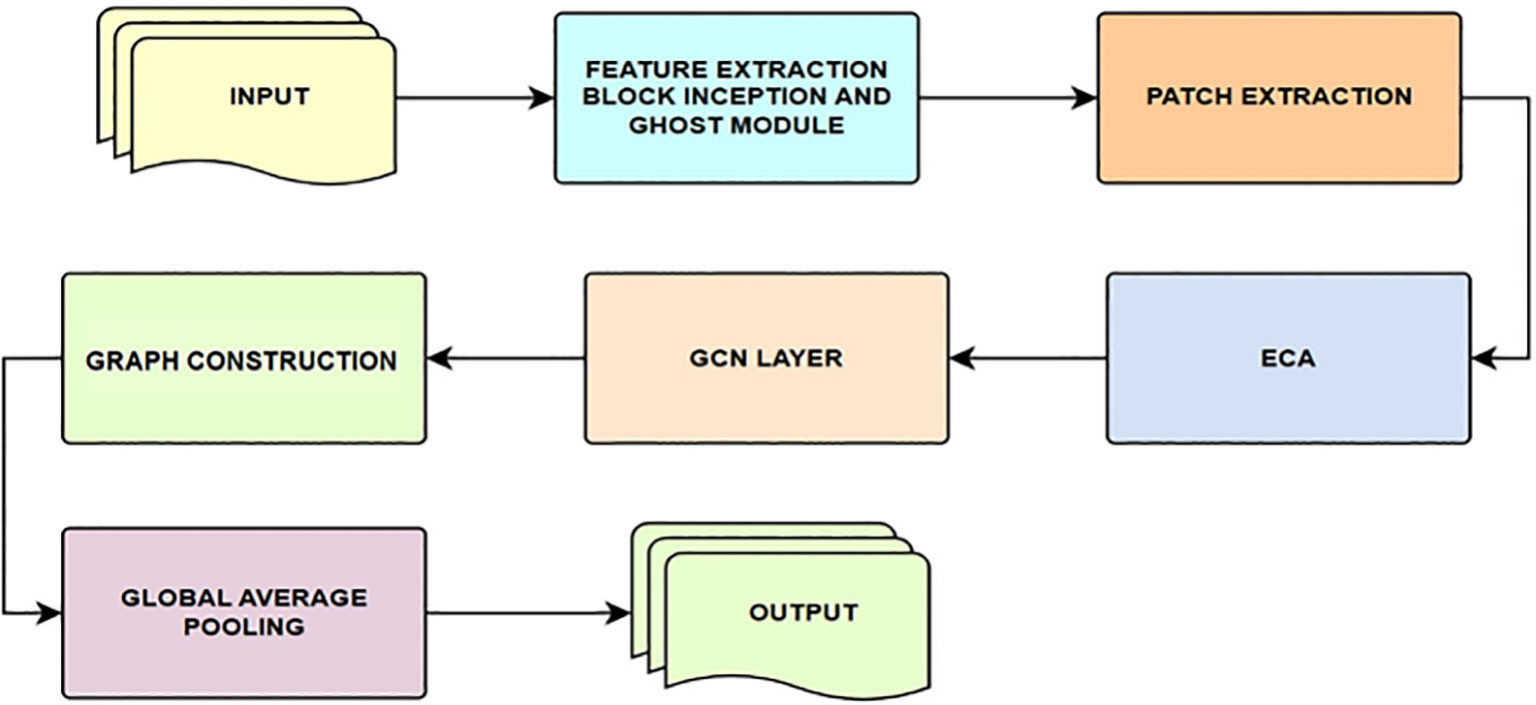
Overview of the GEAFNet architecture.

GEAFNet also proposes a patch extraction method that splits intermediate feature maps into smaller regions for localized inspections of image patches. The patches are then forwarded to a GCN with attention to explicitly model relationships between different spatial regions of the leaf. Graph-based representation allows GEAFNet to model long-range dependencies that convolutional structures fail to capture, allowing for an improved representation of nutrient deficiency. The outputs from the convolutional refinement path and the GCN path are projected back to a shared feature space and then fused. This dual-pathway structure allows GEAFNet to fuse local rich features with contextual global relations, allowing for improved discriminative representations. **Figure 2** shows an illustration of the GEAFNet architecture.

#### 3.2.1 Ghost module

The purpose of the ghost module in the proposed GEAFNet branch is to increase computational efficiency while still maintaining a rich representation of features necessary for classification of nutrient deficiency. Using convolutional neural networks typically leads to redundant feature maps, which incurs further computational cost while not providing a linear increase in feature diversity. To overcome this redundancy, the Ghost module is incorporated into the proposed architecture (Han et al., 2020). Essentially, the ghost module reduces redundancy by producing a small number of intrinsic feature maps with standard convolution and then exploits cheap linear operations to get the additional ghost feature maps for the classifications. A set of intrinsic feature maps is first computed for input $\text{X}\in {\mathbb{R}}^{C\times H\times W}$ as in Equation 4.

(4) $${\text{Y}}^{\prime }=f\left(\text{X};{\text{W}}_{1}\right)$$

where *f*(·) denotes standard convolution with kernel weights W_l_, producing *m* feature maps. To further expand the representation, each intrinsic feature $y_{i}^{\prime }$ undergoes a set of cheap linear transformations Φ*_j_* as calculated in Equation 5).

(5) $$y_{i,j}=\Phi_{j}\left(y_{i}^{\prime }\right),\;i=1,\ldots ,m,\;j=1,\ldots ,s$$

where *s* is the number of transformations per intrinsic map. The final output feature maps are then obtained by concatenation as shown in Equation 6).

(6) $$\text{Y}=\left[y_{1}^{\prime },\ldots ,y_{m}^{\prime },y_{1,1},\ldots ,y_{m,s}\right],\;n=m\times s$$

**Figure 3:**
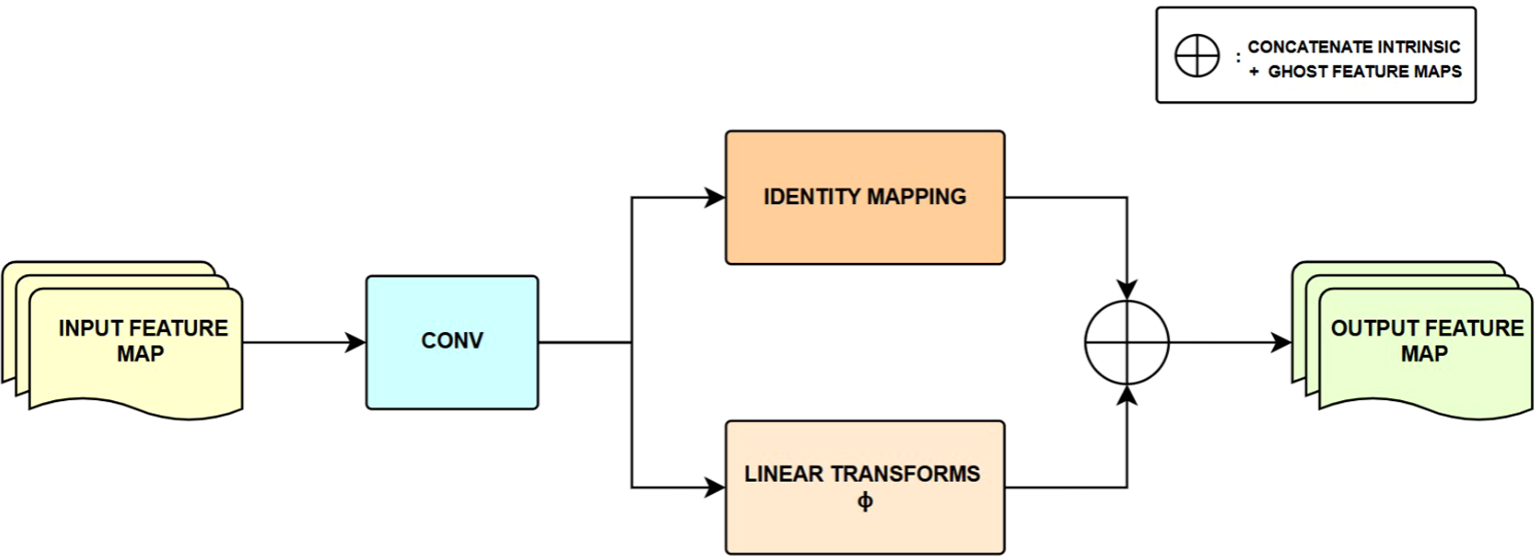
Overview of the ghost module.

This approach closely mirrors the representational capacity of classical convolution but utilizes fewer floating-point operations. The module increases the number of feature maps in convolutional processing areas, which allows an increase in the representational power of the network. The module also improves the efficiency of processing by decreasing the burden while retaining important feature representations. **Figure 3** shows a structural overview of a Ghost module.

#### 3.2.2 Patch extraction

To account for localized structural variations in coffee leaves, GEAFNet includes a patch extraction method. This element splits the intermediate feature maps into non-overlapping regions, allowing the network to model fine spatial patterns. Agronomic indications of nutrient deficiencies will often show the anomaly as localized symptoms. Patch processing ensures that fine-scale characteristics are preserved during global feature pooling (Sohel, n.d).

Specifically, the input feature maps are first reduced in channel dimension through a lightweight convolution, resulting in a compact representation that can be patched.

The feature maps are subsequently applied and divided into patches, and each patch embedding is calculated as an average of the pixels, as mentioned in Equation 7).

(7) $${\text{z}}_{i}=\frac{1}{p^{2}}\sum_{\left(h,w\right)\in \Omega_{i}}{\text{x}}_{h,w},\;i=1,\ldots ,N$$

where x*_h,w_* gives us the feature vector at the spatial location (*h*, *w*), Ω*_i_* gives us the set of pixels in the i-th patch, and N is the total number of patches. This procedure results in a sequence of patch-level descriptors representing localized information on the leaf surface.

Two parallel patch extractors are used, one with a small patch size of 7×7 to highlight fine-scale texture changes and the other with a larger patch size of 14×14 for coarse structural signals. By modeling both granular features and coarse features together, the network is able to elicit a more balanced representation of leaf symptoms. These patch embeddings are then fed to the GCN modules, where inter-patch relationships are explicitly modeled. Patch extraction thereby forms the bridge between convolutional feature learning and graphs, enabling GEAFNet to fuse local characteristics of lesions with long-range spatial dependencies to aid rigor in nutrient deficiency classification.

#### 3.2.3 Graph convolution network

After the patch extraction, GEAFNet applies a graph mechanism to model the relationships between different portions of the leaf. Deficiencies of nutrients in leaves frequently emerge in spatially correlated manners. Symptoms developing in one place in the leaf could be connected to or influenced by symptoms developing in another area. These long-range spatial relationships are rarely captured directly in traditional convolution operations due to their localized receptive fields. Conversely, modeling the leaf as a graph would help the model learn the dependencies between the regions, since graph structures naturally represent spatial and structural dependencies (Zhou et al., 2021).

Given a set of patch embeddings, the GCN first projects them into a latent feature space through a linear transformation as shown in Equation 8).

(8) $${\text{H}}^{\left(1\right)}=\sigma \left({\text{ZW}}_{1}\right)$$

where W_1_ is the learnable weight matrix and σ(·) is a ReLU activation. Pairwise similarities between patch embeddings are then computed to form the adjacency matrix using Equation 9).

(9) $$A_{ij}=\frac{\text{exp}\left({\text{h}}_{i}^{⊤}{\text{h}}_{j}\right)}{\sum_{k=1}^{N}\text{exp}\left({\text{h}}_{i}^{⊤}{\text{h}}_{k}\right)}$$

where h*_i_* and h*_j_* are latent vectors for patches *i* and *j*. This formulation allows each patch to attend more strongly to structurally or texturally similar regions. Feature information is subsequently aggregated information from neighboring nodes, as mentioned in Equation 10.

(10) $${\text{H}}^{\left(2\right)}=A{\text{H}}^{\left(1\right)}{\text{W}}_{2}$$

where W_2_ is a parametric weight matrix that refines node representations. The output is a set of enhanced patch descriptors that incorporate local features and information on context from other regions of the leaf.

The architecture incorporates two GCN branches, a small patch branch (7×7), which encodes fine-grain relational cues, and a large patch branch (14×14), which encodes wider structural dependencies. The outputs of the two branches are reshaped to spatial maps and projected back into convolution space. Subsequently, the multi-level representation is produced by combining small- and large-scale feature maps with convolutional refinement features. This helps the network learn complex local and global relationships while maintaining the classification accuracy.

#### 3.2.4 Efficient channel attention

To refine the feature discrimination process with minimal complexity, the ECA mechanism is incorporated. Nutrient deficiencies often portray minute differences in the intensity of color, vein definition, and texture. These differences may not always be distributed uniformly across all the channels. Hence, the ECA module helps filter out the redundant channels and allows the model to focus on the main features that are important for classification (Wang et al., 2020). The ECA implements a local cross-channel interaction. Specifically, global average pooling is first applied to the feature map $\text{X}\in {\mathbb{R}}^{C\times H\times W}$, yielding a channel descriptor shown in Equation 11.

(11) $$z_{c}=\frac{1}{H\times W}\sum_{i=1}^{H}\sum_{j=1}^{W}X_{c}\left(i,j\right),\;c=1,\ldots ,C$$

The resulting vector $\text{z}\in {\mathbb{R}}^{C}$ is then processed by a 1D convolution with kernel size k, which captures dependencies between each channel and its k nearest neighbors. Subsequently, by performing channel-wise multiplication, the weights are applied to the original feature maps as mentioned in Equation 12.

(12) $${\text{X}}_{c}^{\prime }=s_{c}\cdot {\text{X}}_{c}$$

This process selectively amplifies channels, which represent important signals that portray deficiency-specific cues such as chlorosis or necrosis. In GEAFNet, ECA modules are positioned after both the Inception and Ghost modules and within the mid-level blocks to allow the network to progressively refine the features at multiple scales. ECA enhances the representation to the model and facilitates its ability to capture fine discriminative features from the coffee leaf images.

#### 3.2.5 Coordinate attention

Following the feature extraction from the dual branches, the representations from the DenseNet and the proposed GEAFNet are concatenated to form a unified feature space. The feature maps containing both global structural cues and local fine-grained details are further refined using the CA module to encode spatial dependencies across leaf regions (Hou et al., 2021).

Given the fused feature map $X\in {\mathbb{R}}^{C\times H\times W}$, the CA module decomposes global pooling into two directional encoding operations to preserve positional information. The aggregated features along the vertical and horizontal directions are computed as per Equation 13 and Equation 14.

(13) $${\text{z}}_{\text{c}}^{\text{h}}\left(\text{h}\right)=\frac{1}{\text{W}}\sum_{\text{i}=1}^{\text{W}}{\text{X}}_{\text{c}}\left(\text{h},\text{i}\right)$$

(14) $${\text{z}}_{\text{c}}^{\text{w}}\left(\text{w}\right)=\frac{1}{\text{H}}\sum_{\text{j}=1}^{\text{H}}{\text{X}}_{\text{c}}\left(\text{j},\text{w}\right)$$

where *z^h^* and *z^w^* encode direction-aware contextual information. The encoded features are transformed into attention weights corresponding to each spatial direction and applied to the fused feature map through coordinate-wise modulation, as per Equation 15.

(15) $$Y_{c}\left(i,j\right)=X_{c}\left(i,j\right)\cdot g_{c}^{h}\left(i\right)\cdot g_{c}^{w}\left(j\right)$$

where $g_{c}^{h}$ and $g_{c}^{w}$ represent the learned attention weights along the vertical and horizontal axes, respectively. The CA module helps refine the fused representation by aligning channel responses with their spatial context, enabling the network to focus on deficiency-specific regions across the leaf. The refined features are subsequently passed through global average pooling and the classification head, improving the model’s ability to distinguish subtle nutrient deficiency patterns.

## 4 Results

This section presents the dataset utilized, augmentation techniques applied, and the environmental setup. The section also presents the hyperparameter tuning employed in the study and ablation studies to evaluate the specific tracks within the proposed system.

### 4.1 Data description

The study utilized the CoLeaf dataset containing a sample size of 1,006 images of coffee leaves with nutrient deficiencies (Tuesta-Monteza et al., 2023). The images are categorized into ten different classes: healthy, Nitrogen (N), Phosphorus (P), Potassium (K), Magnesium (Mg), Boron (B), Manganese (Mn), Calcium (Ca), Iron (Fe), and leaves with multiple deficiencies. The dataset provides a range of visual symptoms and different sizes of leaves for various nutrient imbalances in coffee plants. **Table 1** presents the class-wise distribution of image samples across the train, validation, and test splits.

**Table 1:** Summary of class-wise breakdown of the CoLeaf dataset.

Classes	Before augmentation			After augmentation		
	Train	Validation	Test	Train	Validation	Test
Boron-B	61	20	21	284	20	21
Calcium-Ca	103	35	36	339	35	36
Healthy	2	2	2	96	2	2
Iron-Fe	46	10	12	276	10	12
Magnesium-Mg	55	12	13	275	12	13
Manganese-Mn	48	21	20	290	21	20
More-Deficiencies	72	14	13	322	14	13
Nitrogen-N	45	14	15	270	14	15
Phosphorous-P	152	46	47	344	46	47
Potassium-K	57	21	22	268	21	22

### 4.2 Data augmentation

**Figure 4:**
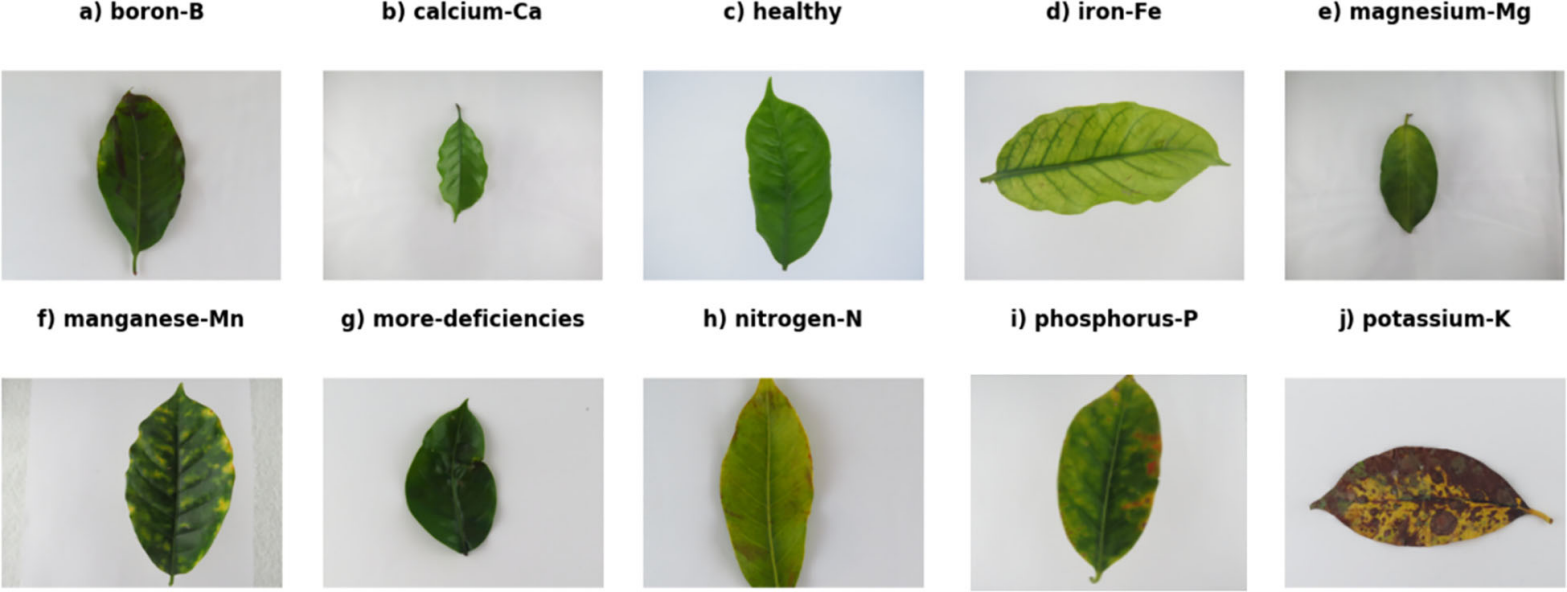
Sample images from the CoLeaf dataset illustrating healthy leaves and various nutritional deficiency symptoms in coffee tree leaves: **(a)** boron (B) deficiency, **(b)** calcium (Ca) deficiency, **(c)** healthy leaf, **(d)** iron (Fe) deficiency, **(e)** magnesium (Mg) deficiency, **(f)** manganese (Mn) deficiency, **(g)** multi-deficiency symptoms, **(h)** nitrogen (N) deficiency, **(i)** phosphorus (P) deficiency, and **(j)** potassium (K) deficiency.

This section presents the data augmentation techniques that were used to balance the image count across all the classes in the training set. The CoLeaf dataset has 1006 images from 10 classes with variation in both size and classes. The training and validation sets were augmented for improved efficiency and minimizing possible overfitting. The following augmentations were applied: (1) Randomly rotated the images 30°, 90°, 180°, or 270° in training and up to a 10° rotation in validation; (2) randomly jittered the brightness, contrast, or saturation. Representative sample images for each of the classes in the dataset are shown in **Figure 4**.

### 4.3 Environmental setup

The experiments were performed on the Google Colab platform. The training and evaluation were sped up using a 16GB VRAM Tesla T4 GPU along with an Intel Xeon CPU. The models were built using the PyTorch framework. The image preprocessing and augmentation stages were accomplished using Torchvision, incorporating basic resizing, flips, rotations, color jittering, and standardization.

### 4.4 Hyperparameter tuning

To increase the efficiency of training, hyperparameter tuning was employed. The learning rates for the backbone and the main network varied between 10^−3^ and 10^−5^, and the weight decay values were in the range of 10^−3^ and 10^−5^. Multiple batch sizes were tested with the comparison of optimizers such as Adam, Stochastic Gradient Descent (SGD), and Root Mean Square Propagation (RMSprop). Learning-rate schedules included Reduce LROnPlateau along with different patience values. In addition, both a standard loss function, cross-entropy loss, and a modified loss function, focal loss, were assessed. **Table 2** presents the summary of the hyperparameters considered and their respective optimal values.

**Table 2:** Hyperparameter tuning results.

Hyper-parameter	Values tested	Optimal value
Learning rate	[10^−3^, 10^−5^]	10^−4^
Weight decay	[10^−3^, 10^−7^]	10^−3^
Batch size	[6, 32, 64]	32
Optimizer	[Adam, SGD, RMSprop]	Adam
Loss function	[Cross-Entropy, Focal]	Cross-Entropy Loss

### 4.5 Ablation studies

This section describes the ablation studies aimed at assessing the individual contributions of the various aspects of the proposed network. The studies tested six configurations: (1) the DenseNet path, as the baseline, to see how it performed as a pure feature extractor; (2) DenseNet with the CA module to enhance that focus on informative spatial and channel-wise patterns; (3) the GEAFNet path to assess graph-based feature extraction; (4) the GEAFNet path with CA and Ghost blocks to assess the effect of lightweight operations and attention; (5) a dual-path model with both DenseNet and GEAFNet to jointly learn distributive features; and (6) the proposed network with DenseNet, GEAFNet, Ghost, and CA modules combined for enhanced representation. Each configuration was assessed with metrics including accuracy, precision, recall, and F1-score to obtain a complete assessment.

#### 4.5.1 Analysis of the DenseNet

**Figure 5:**
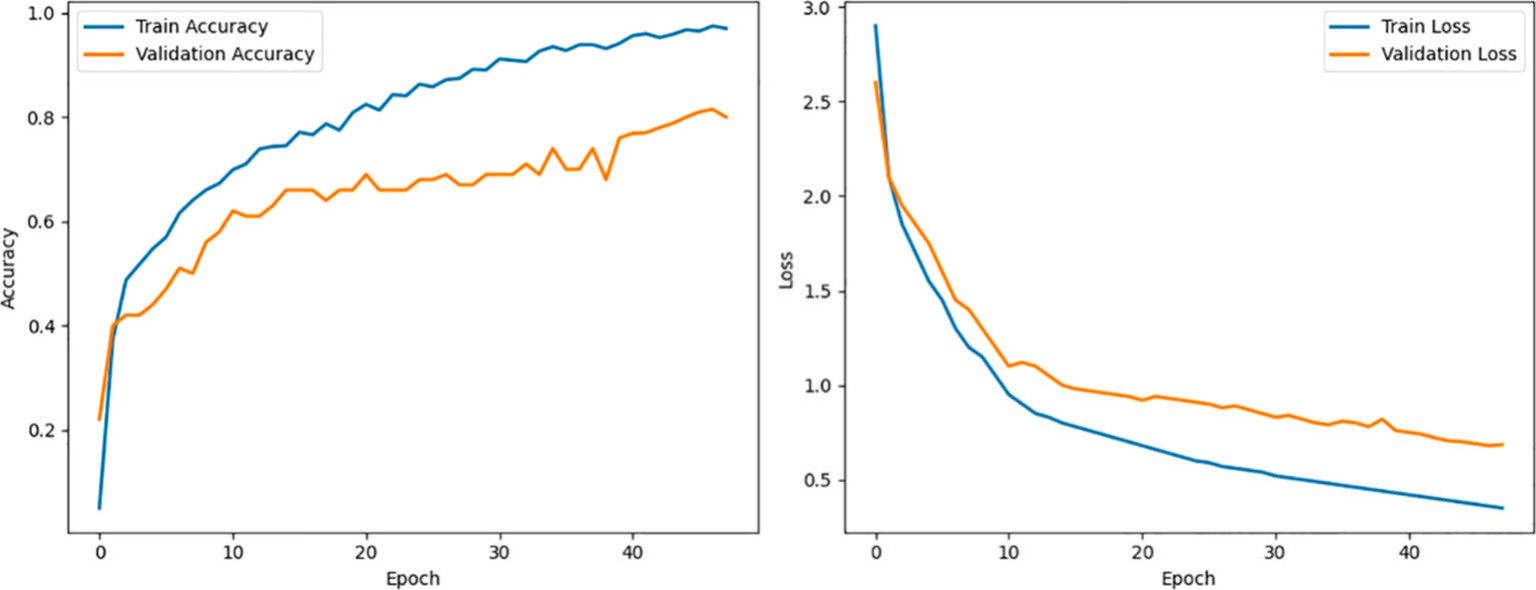
Analysis of the DenseNet.

This section presents the evaluation of the DenseNet to assess its performance for feature extraction and classification in the proposed architecture. The classification head was supplanted with fully connected dense layers along with a global average pooling layer. The model achieved an accuracy of 78.22%, a precision of 76.58%, a recall of 78.22%, and an F1-score of 77.05%. The results present that DenseNet efficiently captures fine-grained structural patterns and local visual features through its dense connection pattern. The results indicate the model’s limitations in focusing on discriminative regions, mild overfitting, and insufficient integration of global contextual information. **Figure 5** illustrates the performance graphs obtained for the experiment.

#### 4.5.2 Analysis of the DenseNet with CA block

**Figure 6:**
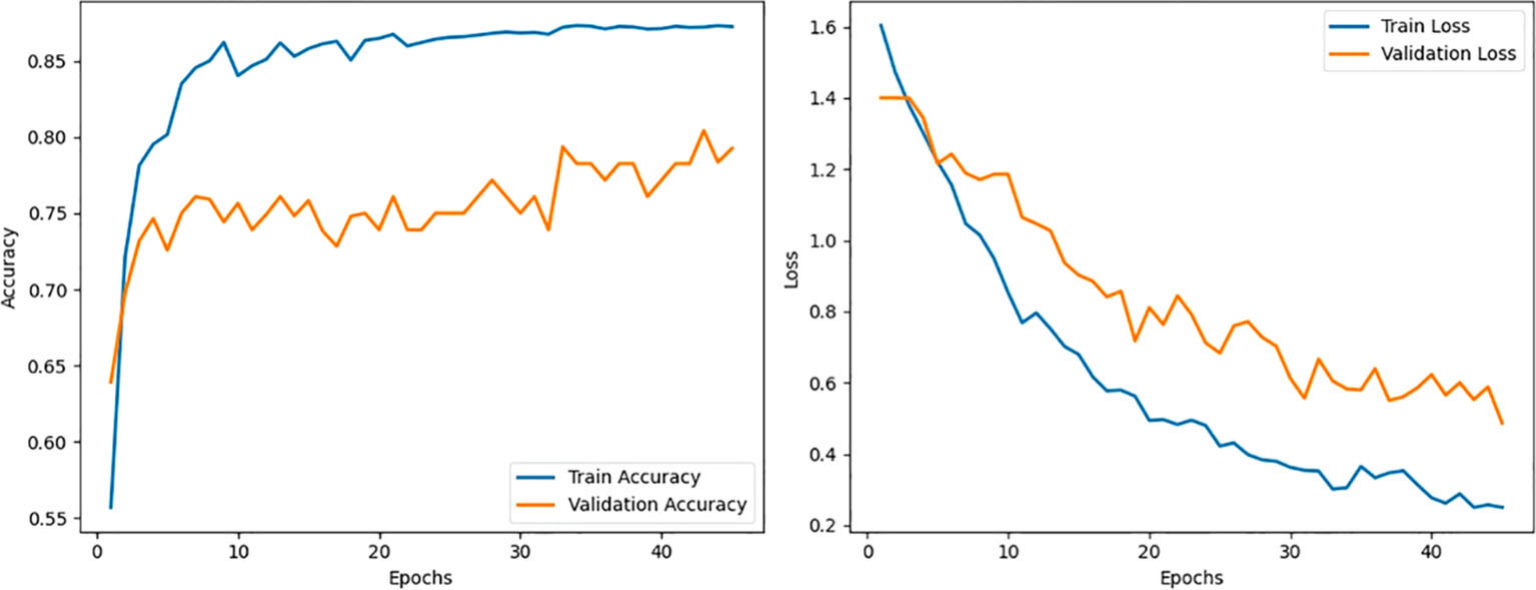
Analysis of the DenseNet with CA module.

This section presents the evaluation of the DenseNet model in addition to the CA module. This helps the models focus on positional information for improved discrimination of the nutrient deficiency features. This modified network achieved an accuracy of 79.41%, a precision of 77.82%, a recall of 79.41%, and an F1-score of 77.72%. These improved performance results highlight the modified network’s ability to attend better to salient structures by adding positional context to the channel attention. **Figure 6** portrays the respective graphs of training and validation.

#### 4.5.3 Analysis of the GEAFnet track

**Figure 7:**
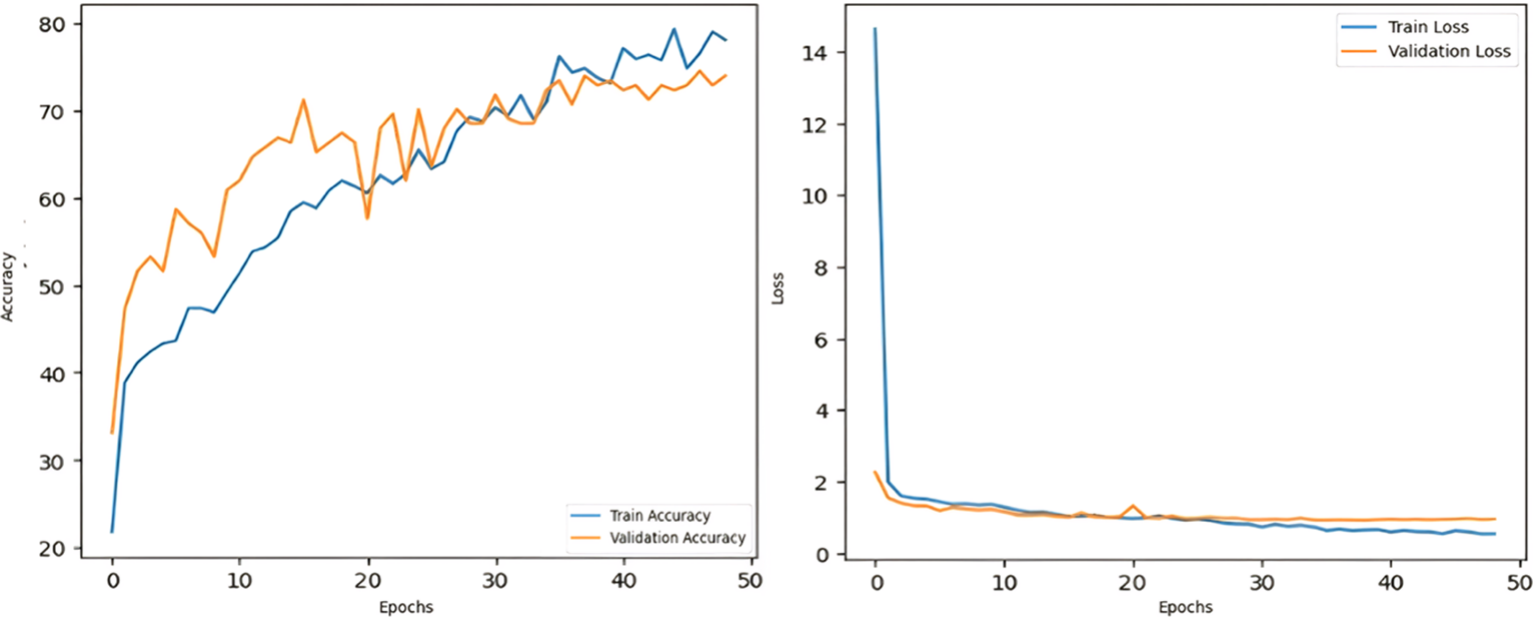
Analysis of the GEAFNet model.

The section assesses the GEAFNet track to evaluate its ability to learn enriched feature representations using graph-enhanced convolutional operations. This track is designed to capture local fine-grained features that complement the global features extracted using the DenseNet. For the GEAFNet track, overall accuracy was 72.83%, precision was 74.32%, recall was 72.83%, and the F1 score was 73.07%. These results highlight the GEAFNet’s ability to model structural relations in the leaf samples and capture richer contextual features. **Figure 7** presents the graphs obtained during the experiment.

#### 4.5.4 Analysis of the GEAFnet with CA and ghost modules

**Figure 8:**
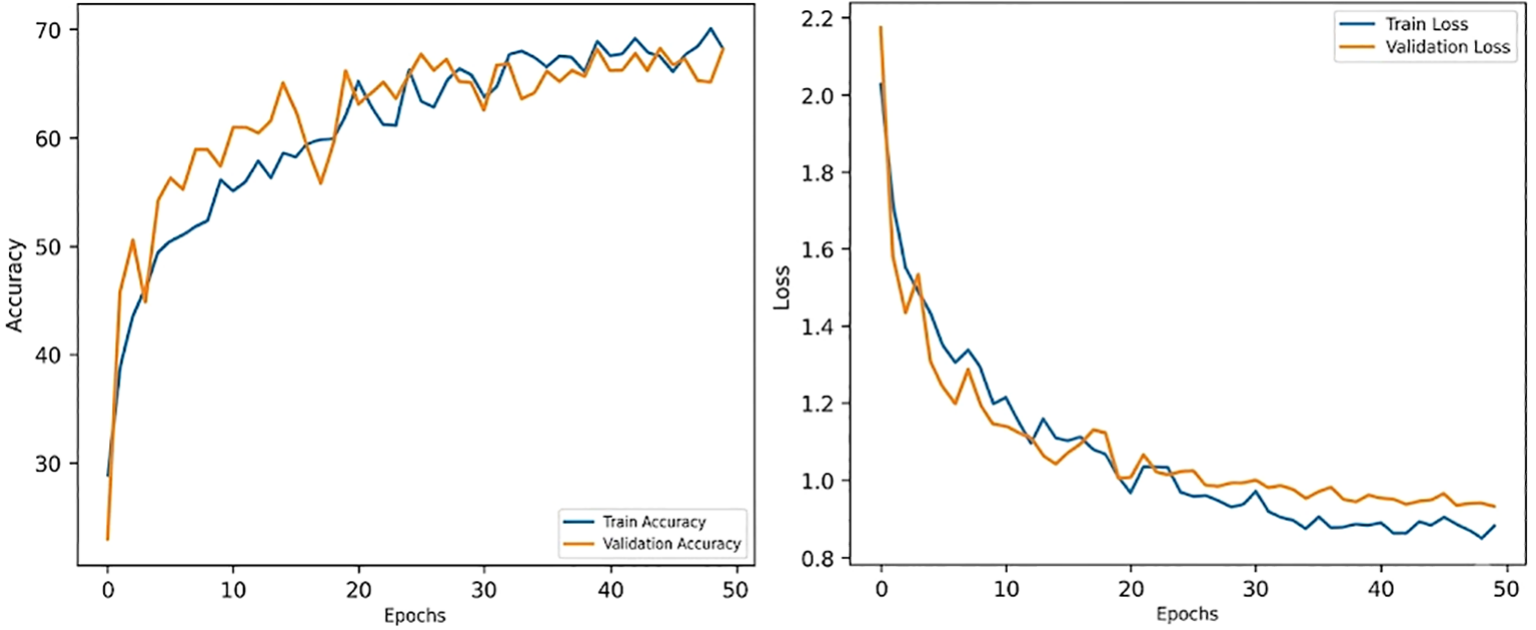
Analysis of the GEAFNet with ghost and CA.

This section presents the evaluation of the GEAFNet with the addition of CA and Ghost modules. The network leverages efficient feature encoding using lightweight convolutional computation and attention-enhanced modules. The Ghost module helps generate additional feature representations with lower parameters and remains computationally lightweight. The CA module allows the network to concentrate on informative spatial channel-wise regions to enhance classification of patterns indicating nutrient deficiency. The improved GEAFNet presented an accuracy of 73.4%, 75.2% precision, 73.22% recall, and an F1 score of 72.87%. Together, these results demonstrate the efficacy of the Ghost and CA modules for learning more improved feature representations while remaining computationally lightweight to accurately classify nutrient deficiency conditions of coffee leaves. **Figure 8** presents the performance graphs obtained in the ablation study.

#### 4.5.5 Analysis of dual-path network with DenseNet and GEAFNet

**Figure 9:**
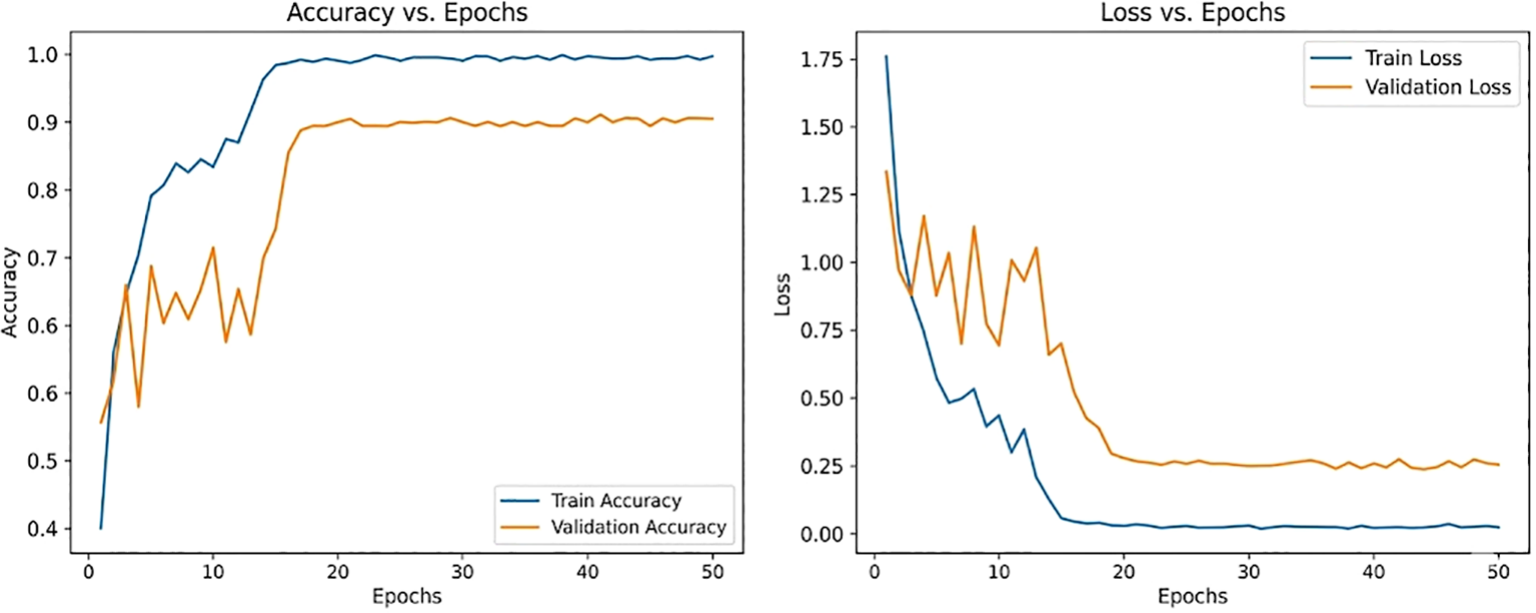
Analysis of the dual-path network without CA.

This section assesses the efficacy of the dual-path network that combines DenseNet and GEAFNet without the addition of the CA block for classifying coffee leaf nutrient deficiencies. The dual-path network utilizes the complementary properties of both networks, with DenseNet providing textural feature extraction and GEAFNet providing relational and graph-based structural features. The feature streams are combined and propagated through the classification layers for the final prediction. The model was trained using the ADAM optimizer, and performance trends of training and validation are shown in **Figure 9**. The dual-path configuration achieved an accuracy of 91.04%, a precision of 91.25%, a recall of 91.04%, and an F1-score of 91.11%. These findings show the effectiveness of the combination of these networks in improving the classification performance when compared to individual models.

#### 4.5.6 Analysis of the proposed CoNutriNet

**Figure 10:**
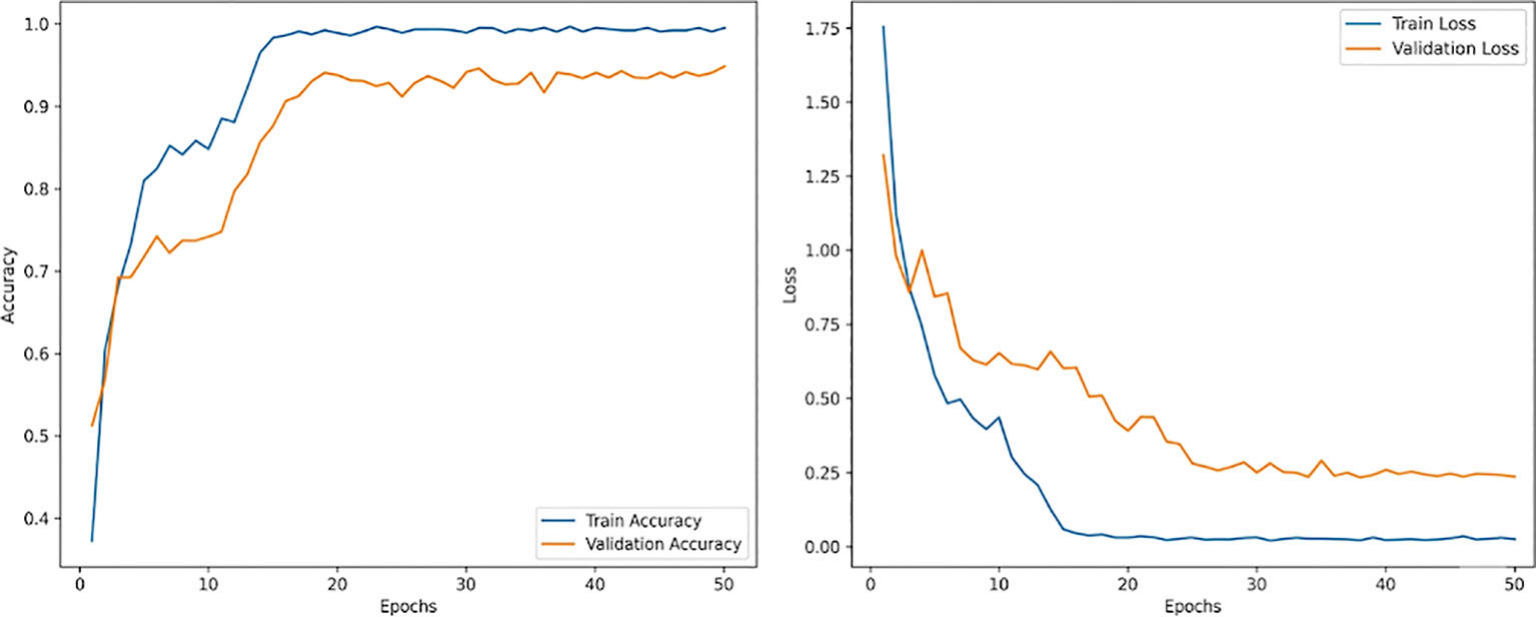
Analysis of the proposed system.

**Figure 11:**
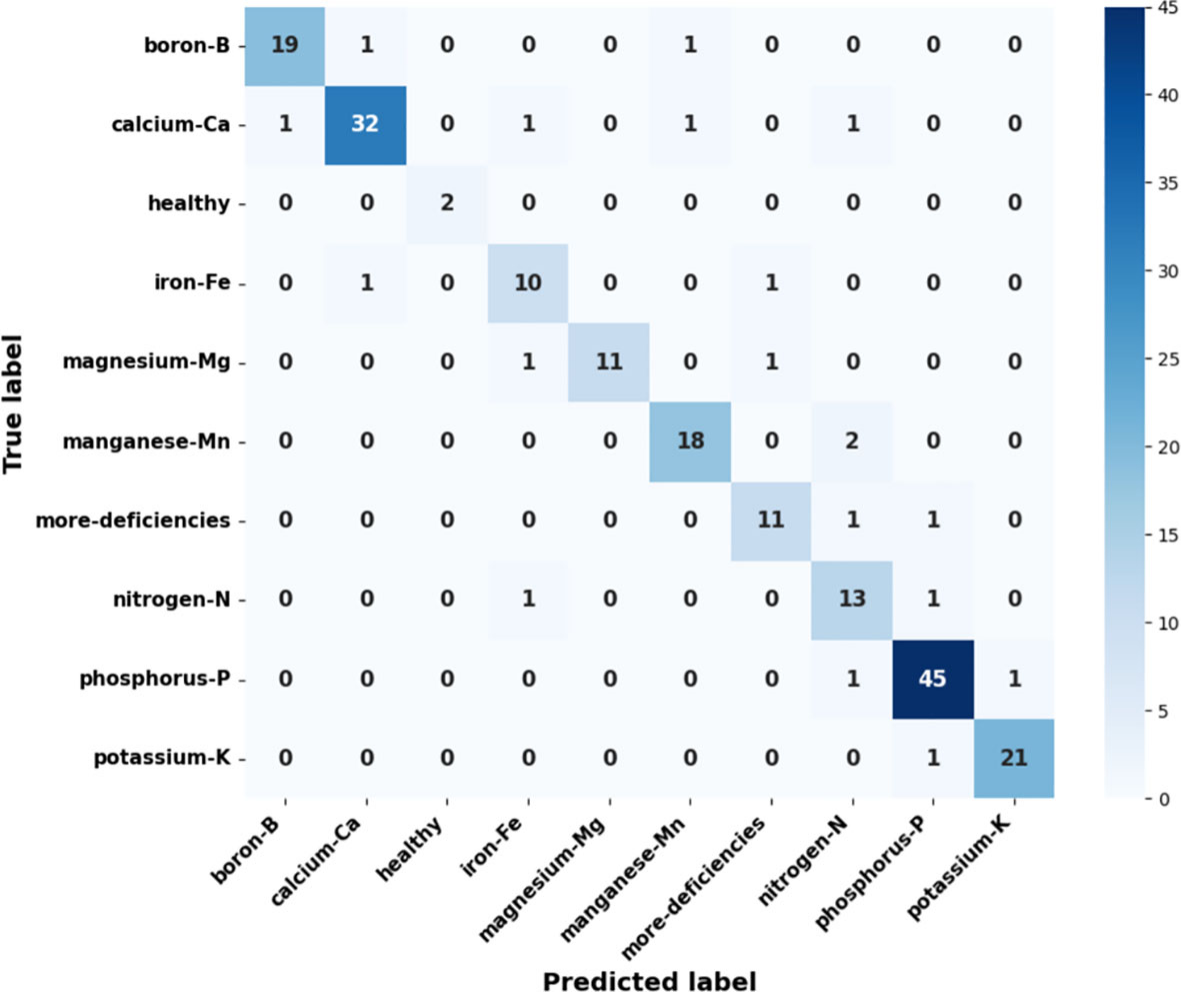
Confusion matrix of proposed system.

This section evaluates the effectiveness of the proposed CoNutriNet that combines DenseNet and GEAFNet through a dual-path approach for the classification of coffee leaf nutrient deficiency. The architecture incorporates both CA and Ghost modules to improve attention mechanisms while maintaining computational efficiency. The architecture incorporates the ability of DenseNet to extract hierarchical global features from the images with the capacity of GEAFNet for relational modeling. The extracted features are aggregated and subjected to the classification layers for final predictions. Training utilized the ADAM optimizer, and the performance graphs are illustrated in **Figure 10**. The hybrid network returned an accuracy of 94.5%, a precision of 94.38%, a recall of 94.5%, and an F1-score of 94.37%. **Table 3** presents the summary of the ablation experiments conducted. **Figure 11** presents the confusion matrix obtained for the proposed system. The matrix indicates that the model achieves strong performance across most nutrient deficiency classes, with a high concentration of correctly classified samples along the diagonal. This reflects the efficacy of the dual-branch architecture in capturing both global structural patterns and local fine-grained features of coffee leaves.

**Table 3:** Summary of the ablation studies.

Ablation study	Accuracy (in %)	Precision (in %)	Recall (in %)	F1-score (in %)	Number of parameters (M)	Inference time (ms)
DenseNet121	78.22	76.58	78.22	77.05	7.22	2.746
DenseNet with CA	79.41	77.82	79.41	77.72	7.32	2.799
GEAFnet	72.83	74.32	72.83	73.07	0.58	0.534
GEAFNet with Ghost + CA	73.4	75.2	73.22	72.87	0.6	0.549
DenseNet + GEAFNet without CA	91.04	91.25	91.04	91.11	9.01	8.347
Proposed Network	94.5	94.38	94.5	94.37	9.45	8.456

Despite the overall high accuracy, a limited number of misclassifications are observed, primarily among classes with visually similar symptoms. For instance, confusion between iron and nitrogen can be attributed to the similarity in chlorosis pattern, where multiple deficiencies produce comparable yellowing effects but differ subtly in spatial distribution. A minor overlap is also observed between manganese and phosphorus, which may be linked to shared characteristics such as patchy discoloration and necrotic regions. Additionally, a few samples from the more-deficiencies class are misclassified into individual nutrient classes. This occurs as leaves affected by multiple deficiencies often exhibit dominant visual traits corresponding to a primary nutrient deficiency, leading the model to favor a single-label prediction. Overall, the observed misclassification patterns are consistent with the inherent visual overlap among nutrient deficiency symptoms rather than shortcomings in the model’s learning capability.

### 4.6 Cross-validation

This section presents the 5-fold cross-validation results obtained from the proposed network to assess the model’s generalization capability. At each iteration, four folds were used for training, while the remaining folds were reserved for validation. This process was repeated five times to ensure that each subset serves as the validation set exactly once. This ensures that the risk of overfitting is reduced and helps provide a reliable estimate of the performance of the model across different partitions of data. The detailed cross-validation results are presented in **Table 4**. A consistent performance across all folds was achieved by the proposed model, demonstrating the stability and ensuring the results are not biased towards a particular partition of the data.

**Table 4:** Summary of 5-fold cross-validation.

Fold	Precision (in %)	Recall (in %)	F1-score (in %)	Accuracy (in %)
1	91.15	91.16	91.18	91.33
2	91.82	91.78	91.81	91.85
3	91.44	91.19	91.31	91.52
4	92.69	92.20	92.55	92.74
5	92.15	92.08	92.10	92.30

## 5 Discussion

This section discusses the comparative analysis of the proposed system with state-of-the-art networks and other research studies. The section also provides the limitations identified about the current proposed system and portrays the future scope of research.

### 5.1 Comparative analysis of the state-of-the-art networks

This section provides a comparative analysis between the proposed architecture and widely used state-of-the-art CNN models for nutrient deficiency classification. Among these baseline architectures, the MobileNetV2 achieved the highest performance with an accuracy of 76.62%. Subsequently, ResNet and XceptionNet reached an accuracy of 71.94% and 71.22%, respectively. EfficientNetB0 and ShuffleNet showed comparatively less performance, achieving accuracies of 70.5% and 65.47%, respectively. The proposed system performed significantly better than all baseline models, attaining an accuracy of 94.5%, along with improvements in precision, recall, and F1-score.

Beyond accuracy, the computational analysis indicates that the proposed CoNutriNet achieves a balanced trade-off between performance and efficiency. With 9.45M parameters and 3.172 GFLOPs, it is considerably lighter than large-scale models such as VGG and transformer-based architectures while remaining comparable to mid-sized networks. The model attains an inference time of 8.456 ms and 118 FPS, which is lower than high-throughput lightweight models like MobileNet but faster than heavier architectures such as Swin Transformer. Despite this moderate computational footprint, the proposed model delivers significantly higher accuracy than all baseline methods, demonstrating its efficacy in model complexity and runtime without reducing classification performance. **Table 5** presents the summary of the comparative analysis with state-of-the-art models.

**Table 5:** Comparative analysis of state-of-the-art networks.

Network	Accuracy (in %)	Precision (in %)	F1-score (in %)	Recall (in %)	Number of parameters (M)	Inference time (in ms)	FPS	GFLOPs (B)
AlexNet	62.38	66.26	63.03	62.38	233	2.09	478	0.710
ConvNeXtV2	63.73	67.51	63.63	61.51	27.91	14.561	69	0.112
ResNet-18	64.36	63.08	61.97	64.36	44.7	2.583	387	1.824
ShuffleNet	65.47	40.26	44.7	50.83	1.0	6.267	160	0.152
VGG-16	67.33	69.54	67.87	67.33	138	8.755	114	15.466
SqueezeNet	69.31	68.7	68.55	69.31	4.78	2.521	393	0.733
EfficientNetB0	70.5	66.63	62.53	62.71	5.3	9.901	101	0.414
XceptionNet	71.22	58.9	57.5	59.25	22.9	5.811	172	4.597
VGG-19	72.28	75.57	73.18	72.28	144	10.542	95	19.628
ResNet-50	73.27	72.42	72.36	73.27	11.4	6.119	164	4.132
RegNetY-8GF	73.73	77.02	71.51	73.73	5.60	9.04	110	0.222
DenseNet-169	75.25	75.26	74.64	75.25	14.32	20.666	48	3.434
MobileNet-V1	75.25	78.88	76.42	75.25	4.3	5.157	195	0.110
ViT	75.25	79.54	76.43	75.25	86	13.367	75	16.848
Swin Transformer	76.24	79.34	77.17	76.24	3	26.134	38	4.372
DeiT	76.24	76.74	74.23	76.24	87	11.333	88	4.241
EfficientNetB1	76.24	80.66	77.59	76.24	30.1	11.306	88	0.609
MobileNetV2	76.62	76.14	76.09	76.62	3.4	5.032	199	0.326
MaxViT	77.82	77.35	78.82	77.58	30.41	28.345	35	0.120
Proposed CoNutriNet	94.5	94.38	94.37	94.5	9.45	8.456	118	3.172

### 5.2 Comparison with existing studies

This section presents the comparative analysis of the proposed system with the existing research works. Existing works mostly rely on conventional CNN models, classical ML algorithms, or hybrid feature extraction networks. Classical ML models incorporated with handcrafted features have shown significant performance, achieving more than 89% in accuracy. The proposed network has outperformed the existing methods with an accuracy score of 94.5%. This result highlights the efficacy of the custom graph-based CNN with DenseNet to capture local and global feature representations. **Table 6** presents the summary of the comparative analysis with existing works.

**Table 6:** Comparative analysis of existing studies with the proposed system on the CoLeaf dataset.

S. no	Source	Model	Accuracy (in %)
1.	Bera et al. (2024)	ResNet-50 with GCN	68.24
2.	Ramirez-Builes et al. (2024)	KNN Classifier	83.5
3.	Diriba et al (Diriba, 2022)	Custom CNN with Decision Tree	85.2
4.	Tuesta-Monteza et al. (2023)	SVM	89.36
5.	Vassallo-Barco et al. (2017)	Shape and Texture with SVM	91.7
6.	Proposed CoNutriNet	DenseNet with Custom Graph-based CNN	94.5

### 5.3 Performance evaluation on external datasets

This section presents the performance of the proposed architecture when evaluated on two external datasets to assess the model’s generalizability. The results highlight the efficacy and adaptability of the proposed network to unseen data, demonstrating its potential in different research and agricultural settings.

#### 5.3.1 Performance on wheat leaf disease dataset

The proposed network was evaluated on the publicly available wheat leaf disease classification dataset (Radowan and Ayon, 2025). The dataset consists of 1,603 images of wheat leaves captured from crop fields in Bangladesh and are categorized into 5 classes: black point, Fusarium foot rot, healthy leaf, leaf blight, and wheat blast. The proposed system achieved an accuracy of 97.52%, with precision, recall, and F1-score of 97.23%, 97.10%, and 97.16%, respectively. This highlights the model’s generalization ability beyond the primary coffee dataset.

#### 5.3.2 Performance on eggplant disease dataset

The proposed network was also evaluated on the publicly available eggplant disease classification dataset (Nirob et al., 2024). The dataset consists of 3,116 images of eggplant leaves and fruits, with high-quality resolution. The images are separated into a number of different diseases, including Cercospora leaf spot, flea beetles, Phytophthora blight, powdery mildew, and tobacco mosaic virus. The proposed system achieved an accuracy of 96.79% with a score of 96.31% precision, 95.63% recall, and 95.96% F1 score.

#### 5.3.3 Performance on rice leaf disease dataset

Additionally, the proposed system was tested on the rice leaf disease classification dataset available publicly in Mendeley data (Hasan et al., 2023). The dataset consists of 1,701 original images of diseased rice leaves categorized into eight classes: Bacterial Leaf Blight, Brown Spot, Leaf Scald, Narrow Brown Leaf Spot, Rice Hispa, Sheath Blight, Leaf Blast, and Healthy Leaf. The images were collected for four months from July 2023 to October 2023 from multiple locations of Sirajganj and Pabna districts of Bangladesh under different outdoor and indoor lighting conditions. The proposed system obtained performance scores of 89.47% accuracy, 88.5% precision, 88.31% recall, and 88.45% F1-score. These results further demonstrate the model’s efficacy in generalization capability across different crop species. **Table 7** summarizes the performance of the proposed network across different crop datasets, showcasing its generalizability to diverse agricultural cases.

**Table 7:** Summary of performance metrics obtained by the proposed system on external datasets.

Dataset	Accuracy (in %)	Precision (in %)	Recall (in %)	F1-score (in %)
Wheat Leaf Disease Dataset (Radowan and Ayon, 2025)	97.52	97.23	97.10	97.16
Eggplant Disease Dataset (Nirob et al., 2024)	96.79	96.31	95.63	95.96
Rice Leaf Disease Dataset (Hasan et al., 2023)	89.47	88.5	88.31	88.45
CoLeaf Dataset (Tuesta-Monteza et al., 2023)	94.5	94.38	94.5	94.37

### 5.4 Limitations and future work

This section covers the potential limitations and scope of future research works in nutrient deficiency classification.

1. Although the CoLeaf dataset establishes a useful benchmark, the small dataset size and imbalanced classes potentially inhibit the model’s generalizability across different nutrient deficiency situations. Some of the nutrient classes are overrepresented, while others have fewer samples, which may affect consistency during classification. An expansion of the dataset with larger and more balanced portions of nutrient deficiencies would help improve robustness and reliability in various scenarios.
2. The RGB imagery may not detect more intricate or early-stage nutrient deficiency symptoms. Limited spectral information in RGB images may result in nutrient deficiencies potentially remaining undetected until they become visually obvious. Future research should utilize hyperspectral or environmental data that may help identify early indications of the nutrient deficiencies.
3. The use of static leaf samples restricts the model’s ability to capture temporal variability in the progression of nutrient deficiencies. Nutrient deficiency symptoms often evolve over time. Incorporating time-series imaging and monitoring the field in a continuous manner may help the system process temporal patterns more clearly and allow early detection in coffee plants.

## 6 Conclusion

Deficiencies of nutrients in coffee plants have adverse effects on plant health and coffee bean production. Conventional diagnostic methods require manual inspection to identify nutrient deficiencies, which is a labor-, time-, and error-intensive process. This study proposes a novel network to address the limitations of nutrient deficiency classification in coffee leaves. The architecture utilizes DenseNet to extract deep hierarchical and global features in parallel with GEAFNet, which helps capture localized fine-grained features. Hierarchical patch extraction combined with GCN is incorporated to enable the model to learn the relational dependencies within leaf regions. This also allows the model to effectively focus on both targeted local cues and contextual relations across the regions within the analyzed leaves.

Finally, the CA module specifically improves the ability of the model to learn positional dependencies and channel dependencies, enhancing the overall classification accuracy.

The multi-scale feature concatenation of both tracks helps in a balanced representation of local and global characteristics, improving classification performance. The proposed CoNutriNet achieved an accuracy score of 94.5% with high precision, recall, and F1 score. These results highlight the efficacy of the proposed system and the advantages of utilizing dense connectivity, attention-guided local feature extraction, and graph-based relation.

## Data Availability

Publicly available datasets were analyzed in this study. This data can be found here: https://data.mendeley.com/datasets/brfgw46wzb/1.
